# Collaboration for Recovery: Expert Mental Health Worker Experiences with the Recovery Assessment Scale – Domain and Stages (RAS-DS)

**DOI:** 10.1007/s10597-025-01476-7

**Published:** 2025-06-04

**Authors:** Hiu Yan Lam, Anne Honey, Justin Scanlan, Nicola Hancock

**Affiliations:** School of Health Sciences, The University of Sydney, Susan Wakil Health Building (D18), Camperdown, NSW 2006 Australia

**Keywords:** Mental health, Recovery assessment scale – domains and stages (RAS-DS), Recovery-oriented practice, Collaborative practice, Mental health workers

## Abstract

The Recovery Assessment Scale – Domains and Stages (RAS-DS) was designed as a tool to promote collaboration between mental health workers and consumers. However, many workers use it solely for its other purpose—as an outcome measure – and lack knowledge and confidence to use it collaboratively. The purpose of this study was to gain an in-depth understanding of the strategies adopted by mental health workers who successfully utilize RAS-DS to facilitate collaborative recovery planning. Using a qualitative descriptive design, semi-structured interviews were conducted with expert collaborative RAS-DS users and analysed using qualitative content analysis. Participants described implementing a range of strategies to facilitate collaborative practice when using RAS-DS. These included strategies to both encourage consumers to complete RAS-DS in a meaningful way and to facilitate discussions about the results for consumer-driven goals and interventions. Findings provide guidance for mental health workers seeking to use RAS-DS collaboratively to support recovery.

## Introduction and Literature Review

According to the World Health Organization (2022), approximately one in eight people worldwide live with a mental health condition. While terminology is contested, in this paper we refer to people with lived experience of mental health conditions who access mental health services as ‘consumers’ (National Mental Health Commission, 2023). Historically, consumers were excluded from decision-making processes about their health and life choices (World Health Organization, 2021). However, in recent decades, there has been an increased call within the mental health industry to embrace consumers’ active participation ( Ness et al., 2014; Viksveen et al., 2022). This has been the result of a consumer-led movement, which advocated a shift in the goal of ‘recovery’ from cure or symptom elimination, to enjoying a meaningful, hopeful and contributing life even in the presence of ongoing symptoms (Anthony, 1993; Leamy et al., 2011). There has, as a result, been an increased focus on recovery-oriented, strengths-based and trauma-informed practice. A recovery-oriented approach recognizes that “each person is the expert of their own life and support should assist them to achieve their hopes, goals and aspirations” (NSW Health, 2022).

Collaboration is a crucial element to recovery-oriented practice (Slade et al., 2008; Symanski-Tondora et al., 2014). Collaboration implies that goals and interventions should be driven, not just by research and clinical evidence, but also by consumers’ aspirations, preferences and values (Castillo & Ramon, 2017; Jørgensen et al., 2024). This is congruent with evidence-based practice, which emphasizes the inclusion of consumers’ values and preferences (Dusin et al., 2023). The adoption of collaborative practice is not only intrinsic to achieving recovery-oriented practice, it can also enhance consumers’ sense of autonomy and control, thereby improving their overall well-being and quality of life (Ventosa-Ruiz et al., 2024). The adoption of collaborative practice is also found to enhance treatment engagement, satisfaction and outcomes that further promote mental health recovery (Abrines-Jaume et al., 2016; Ellison et al., 2018; Gondek et al., 2017; Reist et al., 2022).

The Recovery Assessment Scale – Domains and Stages (RAS-DS) is a self-report tool that was developed to both measure recovery and to facilitate collaboration between mental health workers and consumers on consumer-driven goals. It was developed based on an earlier recovery measurement instrument, the Recovery Assessment Scale (RAS). Although the RAS was found to have good internal consistency and test–retest reliability (Corrigan et al., 2004; McNaught et al., 2007), there were concerns regarding its construct validity (Corrigan et al., 2004). It also showed a ceiling effect (Hancock et al., 2015), hence lacking the sensitivity to differentiate recovery of test takers who were further advanced in their recovery journey. Addressing these issues, removing ill-fitting items and modifying the rating structure resulted in the development of RAS-DS (Hancock et al., 2011).

The RAS-DS consists of 38 items which are grouped into four domains: 1) Doing things I value; 2) Looking forward; 3) Mastering my illness and 4) Connecting and belonging. Currently, RAS-DS has been translated into 18 languages. Formal training and qualifications for its administration are not required, and the instrument and its user manual can be freely accessed through its official website (https://ras-ds.net.au).

As an outcome measure, RAS-DS is used extensively throughout the world. It has been found to be psychometrically sound, demonstrating high internal and convergent validity, test–retest reliability and responsiveness (Hancock et al., 2015; Scanlan et al., 2018). It is also considered to be relevant and easy to use by both mental health workers and consumers (Hancock et al., 2015, 2020). A recent review of 25 self-report outcome measures of personal recovery identified RAS-DS as one of the most promising in terms of its psychometric properties based on currently available data (Felix et al., 2024).

As mentioned, however, RAS-DS was designed as more than just an outcome measure. It was co-designed by consumers and was intended to serve the additional purpose of being a tool to facilitate collaborative and recovery-oriented mental health practice (Hancock et al., 2019). Self-report outcome measures in mental health often show poor completion rates, a key reason for which appears to be workers not valuing these measures (e.g., Boswell et al., 2015). Workers have reported being more likely to use measures that facilitate their work with consumers rather than simply measuring outcomes (Coombs et al., 2011; Trauer et al., 2009). Yet in a review of 99 patient reported and routine outcome measure projects in mental health implemented throughout the world, only 3% had the goal of supporting recovery (Gelkopf et al., 2021). The capacity to use RAS-DS to support recovery-oriented practice appears to be a strength of RAS-DS (Ramesh et al., 2024).

Despite this, many mental health workers have reported using RAS-DS primarily as an outcome measure, and may lack the knowledge or confidence to utilize it for collaboration (Honey et al., 2023). For instance, they have reported difficulties in discussing the results and identifying important areas to focus on with consumers (Hancock et al., 2016; Honey et al., 2023). Indeed, previous international reviews of self-report tools more widely have identified that clinicians’ fear of these tools can be detrimental to practice and rapport building, and can create a barrier to their implementation (Gelkopf et al., 2021).

Although it is clear from research (Honey et al., 2023) and anecdotal evidence that some mental health workers are able to use RAS-DS to facilitate collaboration, there is a lack of research exploring how, in real-life situations, this is done. Therefore, this research aimed to explore the strategies adopted by mental health workers to successfully use RAS-DS to facilitate collaborative and recovery-oriented practice. It aimed to provide insights and guidance to mental health workers who wish to use RAS-DS, or any other self-report recovery-focused tool, collaboratively to support consumers’ active engagement in self-assessment and recovery planning.

## Methods

### Design

As this study aimed to gain insight into a poorly understood topic – the strategies employed by mental health workers when using RAS-DS to promote collaborative and recovery-oriented practice – a qualitative approach was adopted (Hammarberg et al., 2016). Specifically, a qualitative descriptive study was used as this study aims to provide readers with direct description of the topic rather than, for instance, building theory (Sandelowski, 2000). Ethics approval was obtained from The University of Sydney Human Research Ethics Committee (Protocol #2023/814). The Consolidated Criteria for Reporting Qualitative Research (COREQ) guided the reporting of this study (Tong et al., 2007).

### Recruitment & Participants

To ensure the recruitment of individuals most likely to yield appropriate and important perspectives, purposive sampling was employed (Kelly, 2014; Mason, 2002; Trost, 1986). Specifically, the key informant technique was used. Key informants are individuals with “unique access” to the phenomena being studied (Hughes & Preski, 1997; Tremblay, 1957). They possess the knowledge and ability to meaningfully synthesize their experiences, thereby offering rich and reflexive insights (Marshall, 1996; Payne & Payne, 2004; Tremblay, 1957). Therefore, the only inclusion criterion in this study was that participants were known to be expert users of RAS-DS. Expert users were defined as people who routinely utilized RAS-DS as a tool to facilitate collaborative practice and consumers-driven recovery planning.

Potential participants were identified from a pool of mental health workers known to the RAS-DS developer (author 4) through previous communications around its use. Author 4 sent out email invitations including a Participant Information Sheet and consent form. To minimize any potential pressure and/or coercion to participate, participants were asked to return the consent form directly to author 1 and were assured that author 4 would not know who participated. Author 4 sent a total of 13 invitations, leading to interviews with six participants. Four additional participants were recruited through snowball sampling, which further expanded the study’s reach as key informants were knowledgeable of others with similar expertise (Pahwa et al., 2023). Recruitment ceased when no new concepts were emerging from the data and the major categories were well-understood, indicating data saturation. This is a common and appropriate way of determining sample size in qualitative research (Fossey et al., 2002).

### Data Collection

Data were collected through online interviews via Zoom between February and August 2024. Semi-structured interviews were conducted to provide participants with ample space and opportunity to express their ideas and opinions (Cohen & Crabtree, 2006) so as to contribute to the richness of data required for a qualitative descriptive study (Bradshaw et al., 2017). Interviews lasted between 60 to 100 min, with an average of 72 min.

An interview guide was developed from literature and the experiences of the RAS-DS research team (Kitto et al., 2008). It included questions about participants’ demographics, use of RAS-DS, and strategies for using it to facilitate collaboration with consumers. All questions within the interview guide were single-faceted and open-ended to facilitate spontaneous and in-depth answers (Ryan et al., 2009). Follow-up questions were asked throughout the interviews to seek clarification of meanings and further expand upon participants’ viewpoints to gain greater understanding of their experiences (Holloway & Wheeler, 2010; Whiting, 2008). As is appropriate with qualitative interviews, the interview guide was modified throughout data collection to collect richer data and obtain further data relevant to the emerging analysis (Charmaz, 2014; McGrath et al., 2019). The initial interview guide is available in Supplementary file 1.

Interviews were audio-recorded with participants’ consent and transcribed verbatim. This minimised researchers’ recall bias, and eliminated interruption to interviews due to constant note taking. Any information that could potentially result in the identification of participants was removed during the transcription process. Concurrent data collection and analysis allowed ongoing refinement of the interview guide and determination of the point at which data saturation was reached.

### Data Analysis

Qualitative descriptive studies require researchers to describe data in a manner that closely resembles participants’ own language (Sandelowski, 2000; Sullivan-Bolyai et al., 2005). Hence, qualitative content analysis, which focuses on extracting categories and meanings from data, but not on developing a theoretical framework (Cho & Lee, 2014), was adopted to analyse the data collected. Both the manifest and latent content of data were examined and interpreted inductively through line-by-line open coding (Hsieh & Shannon, 2005). This involved codes being developed based on the data obtained rather than from researchers’ pre-conceived ideas (Cavanagh, 1997; Kondracki et al., 2002), ensuring participants’ views and voices were prioritized, and all relevant data considered (Drisko & Maschi, 2015; Holton, 2008). During this process, the researcher examined each chunk of data (such as a sentence or statement) to identify the underlying concepts. Code names were then developed to summarize the concept. For example, the statement “I talk to them about that this is just a way for me to get to know them and for me to understand their views, and what they're feeling about their life” was initially coded as “RAS-DS helps staff understand consumers and their viewpoints”, “introducing the RAS-DS”, and “explaining the purpose of RAS-DS”. The first two interview transcripts were independently coded by both authors 1 and 2. Codes were then compared and discussed to reach consensus. The remaining data were coded by author 1 with regular meetings with the research team to discuss, question and revise the codes. Codes were then compared for similarities and differences and grouped into sub-categories and, finally, categories through discussions between the authors. The software NVivo 12 was used to assist in managing the codes generated.

## Findings

Ten expert RAS-DS users participated in the study. They had an average of 13.3 years of experience in the mental health sector. They came from two countries and five mental health disciplines. Their experience with RAS-DS ranged from approximately 8 months to 6 years, with an average of just under 4 years. An overview of participants’ demographics is presented in Table 1.

**Table 1:** Participants’ demographics

Characteristics		N
Gender	Female	6
	Male	4
Occupation	Occupational Therapist	2
	Peer Worker	3
	Rehabilitation worker	3
	Social Worker	1
	Support Worker	1
Country	Australia	7
	Canada	3
Duration of practice in mental health	0 – 5 years	2
	6 – 10 years	1
	11 – 15 years	3
	16 – 20 years	1
	20 + years	2
	Did not disclose	1
Duration of RAS-DS experiences	0 – 2 years	2
	3 – 5 years	7
	5 + years	1
Practice setting	Community	9
	Inpatient and community	1

All participants reported offering RAS-DS to most or all consumers and that the vast majority completed it, with estimates ranging from 70%—90% for the first completion and 90%—100% for subsequent completions. One participant reported that, in their organization, offering RAS-DS was mandatory, while four reported that they were “highly encouraged/recommended” by their organization or program to do so. For the remaining five, offering RAS-DS was at their discretion.

Participants described implementing a range of strategies to facilitate collaborative practice when using RAS-DS. These included strategies to both encourage consumers to complete RAS-DS meaningfully and to facilitate collaborative discussions about the results. The final coding framework is summarised in Table 2 and described in detail below.

**Table 2:** Coding tree for facilitating collaborative practices

Facilitating meaningful completion	Choosing appropriate timing	Evaluating consumer readiness
		Building rapport and trust
	Appropriate RAS-DS orientation	Clarifying the purposes and benefits
		Clearly indicating what to expect
	Tailoring administration	Providing options for completion
		Additional guidance where needed
		Creative completion strategies
Discussions based upon RAS-DS results	What to discuss	High scores
		Low scores
		Changes over time
		Aspects highlighted by consumers
	How to discuss	Strategic use of diverse communication strategies
		Giving consumers’ views and opinions precedence
		Providing a safe and comfortable environment
		Being sensitive and strengths-based
		Adopting a trauma-informed approach

### Facilitating Meaningful Completion

To maximize consumers’ willingness to complete and engage meaningfully with RAS-DS, participants reported offering RAS-DS at a suitable time, providing appropriate orientation, and tailoring administration to consumers’ needs.

1. Choosing the appropriate timing

Offering RAS-DS at the right time was seen as critical. As P4 explained “*you pick your moments, because you also want people to give a nice, an honest reflection of where they’re at*”. To aid their decisions, participants reported considering both consumers’ readiness and, particularly for the initial completion, their own level of rapport with consumers.

Participants evaluated consumers’ readiness by considering both their circumstances and whether they were “*in a head space to deal with [an] assessment*” (P7). They did this through reflecting on pre-assessment conversations and their knowledge of consumers. For instance, many reported they would not offer RAS-DS if consumers were acutely unwell, in crisis or distress, or had pressing needs that needed to take priority. P7 gave the example that “*I’m not going to offer a crying person a RAS-DS [because it would be] like I haven’t listened to you for the last hour*”. P9 summed up the timing issues by saying “*I’ll only sort of work with them on a RAS-DS if I know that they're in a position of comfort and safety and ready to talk about recovery*”. For subsequent completions, some participants were guided by organizational guidelines, such as to complete it every 6 months or midway through the program, while others waited until natural turning points, such as when recovery work on a particular area was completed or they sensed that consumers had made progress in their recovery. All participants, however, noted that being flexible and using their judgement about what was appropriate were essential. For example, if “*there may be a clear goal that you're working on with someone, and they're still working on that… you wouldn't kind of come back to a RAS-DS at that point*” (P5).

Many participants (n = 7) also emphasized the importance of building rapport and trust before offering RAS-DS. If a consumer declined RAS-DS initially, participants would often re-offer it later if they thought the consumer’s circumstances or attitude may have changed, or better rapport had been built. For example, P7 often found that consumers “*feel differently about it later*” and “*because of the relationship I've built, they're happy to do it*”.

The decision of when to offer RAS-DS was always, however, an individual one. As P10 described: “*Some people who come in just don’t have the scope or the space for that, but others are really looking at how do I get this ball rolling*”. P4 agreed, saying that even though they would not usually offer RAS-DS to people who were acutely unwell: “*I've… had people who are acute who love paperwork, who can't wait to answer questions. So, really, it's unique with the person, and I really think you can't categorize*”.

2. Appropriate RAS-DS orientation

Participants emphasized the importance of taking the time to provide consumers with appropriate orientation to RAS-DS. In P8’s experience, “*you can have a thorough explanation in five minutes of what this document is. So, when I say dedicate time, I mean make it intentional*”.

Different workers introduced RAS-DS differently, but two aspects were emphasized. First, participants felt that it was crucial to clarify the purposes and benefits of RAS-DS completion. As P8 explained: “*We want to be able to make it very clear why they're doing it, who they're doing it for, and how does this help them stay on track with their recovery”.* They emphasized the role of RAS-DS in both supporting consumers’ self-exploration and giving mental health workers deeper insights into consumers’ perspectives, facilitating holistic and person-centred support. P9, for example, reported telling people that: “*it’s a good chance for you to really understand where you're at and it’s also a good chance for me to get a better understanding of you, so then we can work together to build some recovery goals that are important for you, that you really want to develop and work on*”. Several emphasized that RAS-DS completion was not for the service, but for the consumer themselves, and felt that this would distinguish RAS-DS from the many other assessments consumers were often asked to complete. This was seen as particularly important for consumers who had past negative and even traumatic experiences with mental health services. Participants who were peer workers would also share their personal experiences of RAS-DS usage, illustrating how it had benefitted them. For instance, P3 described: “*sharing your own journey of using the tool or your own experience can help a person to rethink how they're using this tool, how fast they're going through it, and how they can gain more from it*”.

Second, participants provided consumers with clear indications of what to expect. For instance, they explained and showed consumers the format of RAS-DS, saying things like “*it just asks you all these overarching statements, and then you can kind of rate how you feel you're going in those*” (P1). They allowed ample time for completion, ranging from 5 to 60 min depending on consumers’ needs, and were explicit that “*we want you to take time and really reflect where you’re at*” (P8). Some would be upfront with consumers that “*some of the questions are difficult*” (P9) and may even cause discomfort. Critically, participants emphasized in their orientation that answers would be discussed afterward. For example, P5 reported always telling consumers:

*“At the end, we'll have a chance to, you know, explore this, any of the domains in more detail, or any of the questions. So, you know, if you want to make any notes, you can, and [that] gives us a chance to kind of discuss this together.” (P5)*

3. Tailoring administration

Participants suggested that, given each consumer is unique, how RAS-DS was completed and the support provided should be specific to consumers’ needs. This included providing consumers with suitable options for engaging with RAS-DS. This was important not only to enable consumers to complete RAS-DS in their preferred ways, but also to empower them with decision-making opportunities and give them a sense of safety. First, participants emphasized that whether to do RAS-DS should always be up to the consumer and never be mandatory. Several also reminded consumers that they could skip statements they were not comfortable answering. Other options included varying the: completion format (pen-and-paper or digital); location of completion (at home, within the community, or in consultation rooms); pace of completion (in one session or multiple sessions, with or without breaks); and support for completion. P1 described some of the options they gave:

*“I would show them the piece of paper, and I would say to them like, ‘Do you want to take it and do it? And then we can meet up and at another time? Or do you want to do it now?’... I also offer whether they want me to read it, like read the statements out to them, and then they can just verbally give me their rating. Or if they wanted a piece of paper, so I always take two… and then I read it out, and then they just write their own answers, but I just sit with one and I read it out. I give them a few options.” (P1)*

When working with consumers who might experience reduced cognitive function or low English literacy, participants would often provide additional guidance to support these consumers in completing the RAS-DS. For example, they might help break down or paraphrase statements that the consumer was struggling with or provide additional tailored prompts and explanations using examples. Participants also reported various strategies for if a consumer had trouble deciding which score to assign. Some participants reported clarifying or providing some guidance. For example, P8 said “*sometimes it does just require a quick conversation, like just to have them explore it out loud and have you as a sounding board and then they kind of get to that answer themselves*”. Others reassured consumers “*not to over-think… [as] there’s no wrong answer or right answer*” (P7) and to “*just read it, first response, good response*” (P10) as follow-up conversations would be conducted to delve deeper anyway. Participants utilizing RAS-DS + (a version of RAS-DS that includes spaces for comments) often advised consumers to just note their thoughts in the comments sections to be discussed afterwards.

Participants described various other creative completion strategies, especially for consumers with cognitive issues or for whom English was a second language. Some found completion over multiple appointments useful to ensure ample time for thorough explanations. At times, strategies like "*looking at it together… like reading [the statements] out together*” (P5) or "*hav[ing] another staff read the questions to the person”* (P3), were also thought to help. When working with consumers with limited English literacy, some participants were able to use the person’s first language, either by providing a translation from the RAS-DS official website or translating each statement themselves. For instance, P2 overcame language issues by “*just putting the [translated version] beside it, and then the English, and then they were able to answer*”.

### Discussions Based upon RAS-DS Results

According to participants, when using RAS-DS to facilitate collaboration, the conversations that happened after RAS-DS completion were the most critical part. These discussions could take between 10 and 60 min, an investment participants deemed well worthwhile. For instance, as highlighted by P2, “*there's no time wasted, and it doesn't impede in the work, and it actually just enhances the work*”. Participants used judgement and knowledge of the person in all discussions and reported that it was important to consider both what to discuss and how to discuss it.

1. What to discuss

Participants described different approaches to structuring their discussion. While some anchored most of their discussions around individual statements, others tended to look at domain scores. The main foci of discussions were high scores, low scores, changes over time and aspects highlighted as important by consumers.

Discussing areas in which consumers scored highly was seen as important, as P9 explained, “*being strength-based, you know, [I] often couch it in those areas where they're completely true or mostly true and sort of teasing out the things that are working for them, the things that are good in their life*.” Low scores, on the other hand, had the potential to suggest areas that consumers might want to work on. For example, P1 describe how “*if they've rated really low on one, I'll ask them those questions like… ‘What do you think is happening there?’… ‘Is this, you know, something that you would like to see kind of move up in your rating?*’”.

On subsequent completions, participants would also discuss consumers’ current scores compared to previous scores. This was thought to be beneficial in illustrating consumers’ progress and achievements in an impactful manner, as P8 stated: “*when they are doing these markings, they can really see that change, so I've definitely had participants who are shocked by the jump in numbers*”. While some participants just compared the numbers, others provided graphs to help consumers visualize their changes in scores and progress. Participants suggested that discussing the changes in scores helped further facilitate explorations of consumers’ strengths, things that had changed in their lives, and new areas they would like to work on. P4, for example, described their process:

*“I actually take the results with me when they do another one, just to say, ‘Well, last time you did it… this is where you were sitting, and this time’, if, if things are worse or better, you know, ‘what's changed, what's working for you? What's different this time?’. Particularly if it goes down, like, you look for, ‘What's happening for you? What are the life events around you that's influencing how you're feeling about yourself at this time?’. And you know, and that may, maybe we bring a new goal out of that, that they want to work on something.” (P4)*

While high, low and changing scores were good ways to start conversations, participants highlighted the importance of discussing the areas that were most meaningful to consumers. For instance, participants using RAS-DS + reported discussing statements to which a consumer had added comments. Others encouraged consumers to highlight statements that confused them, were of particular importance to them or that they would like to discuss further by either suggesting they “*put like a little star beside [the statement*]” (P8), or asking as part of the discussion, “*what are your thoughts… Is there anything that stands out for you? Is there anything that surprised you with how you answered a particular question?*” (P4). Participants also expressed the importance of paying particular attention to discussing statements that evoked emotional responses in consumers. P6, for example, said that “*sometimes when you're asking questions… they can get saddened… But it's having that discussion, right, being able to acknowledge that this might be something that we want to work on together and keep building on*”. Participants agreed that not shying away from these conversations was critical:“*It doesn't frighten me, because I think if it's out there, and you have that relationship, then you know, if someone's becoming upset or it's kind of brought up a conversation about how hard that is for them, well, I think that's a good thing. That's our role is to be there with someone through those kind of hardships*.” (P5).

2. How to discuss

To facilitate their conversations with consumers, participants reported using a number of strategies and skills. These were not unique to using RAS-DS but were seen as an integral part of working with consumers in a trauma-informed and recovery-based way.

First, participants emphasized the strategic use of diverse communication strategies as critical. For example, they highlighted the importance of: using non-leading and open-ended questions; active and reflective listening; being “*attentive to the body language*” (P7); adopting an “*informal and conversational*” style (P5); adjusting language and expanding questions based on consumers’ circumstances, needs and “*what they might say to me”* (P1); validating and normalizing consumers’ experiences and feelings; being non-judgmental; being honest and transparent; and showing empathy. P2, for example, pointed out that “[*what] we want to really be mindful of is to not influence somebody's response, or perceive any judgment, or even just our tone*”, while P7 explained that “*I can’t tell somebody that I understand what it's like to be a voice hearer but I can empathize with the distress of and the anxiety and maybe the depression it causes*”.

To further facilitate collaboration and avoid power imbalance, participants ensured consumers’ views and opinions were given precedence during conversations. This included providing consumers with the freedom to decide the discussion’s topics and flow, as well as the goals or areas that they want to prioritize. For instance, as highlighted by P8, “*it comes back to choice, because it’s not our role to get them to a higher place. It’s their choice that if they want to interact with something… We [would] say, ‘do you want to do some work on this’, and then they can choose to say yes or no*”. One peer worker, who emphasized how important it was in their role that interactions were led by each consumer’s immediate agenda, described how, rather than formally going through specific questions directly*,* they would instead "*look at the lowest scoring things and make a note that these things are troubling to that person or they don’t feel good about them and I will bring them in in an organic way in our conversation*” (P7).

While all participants agreed that safe and comfortable environments were critical for facilitating discussions, their opinions about what constituted such an environment differed. Some considered that conducting discussions in the community helped reduce clinical ambiance, making consumers feel more at ease. However, others thought that these discussions should be conducted more privately. For discussions in the community, participants reported choosing an appropriately private venue with no “*strong distractions or people around, so… we can fully discuss the content of what we’re going to be going over*” (P8). Some participants adjusted their sitting position in relation to consumers, like “*try[ing] to move and sit with them, so the paper’s in between us*” (P1). Participants who were peer workers would also share their personal mental health experiences to further help consumers “*feel more comfortable then, sharing something*” (P7).

Some participants highlighted the need to be sensitive and to remain strength-based (focusing on consumers’ strengths and resources (Tse et al., 2016; Xie, 2013)). This was particularly important when discussing areas where consumers’ scores were low or had decreased over time. For instance, they would reassure consumers that it was okay to experience a decrease in scores because recovery is not a linear process and that “*you're not unlearning the work that you’ve done and you don’t lose that recovery… you're just going through another area of recovery*” (P8). They emphasized that downward fluctuations could be due to a range of issues, not all of which were negative, and invited consumers to consider these possibilities. For example, consumers may just be “*getting to know yourself more once you start interacting with recovery-based work*” (P8), or may be feeling more comfortable being frank with the mental health worker as rapport strengthens. They may also be coping with changes to “*external factors out of your control*” (P8) that would naturally lead to a decrease in scores. P3, a peer worker, explained how even if “*things can be not very good, or somehow it's unpacked in that way, then you can still use it to transform and share elements of your own journey, and others’ journeys as well and provide, hold hope for people*”. A couple of participants also noted that they used their judgement to decide what to highlight and may not bring something to consumers’ attention if it is “*potentially going to do some harm*” (P9).

When discussing areas that had brought up unpleasant emotions within consumers, participants emphasized the importance of adopting a trauma-informed approach. A trauma-informed approach recognizes the widespread impact of trauma and seeks to avoid re-traumatizing consumers by prioritizing principles like safety, trustworthiness and empowerment in all services (Trauma-informed care implementation resource center, 2025). For instance, rather than insisting consumers discuss topics they may not be ready to share, participants said that they would wait and continue to build rapport as “*over time, our participant will open up to me, and then will start telling me a little bit more… [then] I can pick up on something and inquire*” (P6). If a consumer shared their unpleasant experiences, participants were particularly careful to probe gently, “*and really being guided by someone in what they do want to talk about*” (P5). They also checked in with consumers at the end of session to ensure they felt safe and positive before leaving.

## Discussion

This is the first study to examine how expert RAS-DS users use the RAS-DS to facilitate active collaboration and recovery-oriented practice with consumers in the real world. Participants reported strategies to encourage consumers to complete RAS-DS in a meaningful way, including choosing the appropriate timing, providing adequate orientation to RAS-DS and tailoring their administration style to consumers’ individual needs. To facilitate productive discussions about the results and thus consumer-driven goals and interventions, they asked questions about items and domains in which consumers showed high and low scores or changes over time, or expressed an interest in exploring. They used a range of communication strategies, gave consumers’ perspectives precedence, ensured safe and comfortable environments and were sure to be sensitive and strengths-based during discussions.

These strategies not only support but further expand upon and provide details on some of the “active efforts” that participants in a previous study suggested might facilitate the collaborative use of RAS-DS with consumers (Honey et al., 2023). These included ensuring consumers’ comfort and being flexible. This study has also identified several strategies that mental health workers could adopt to address some of the challenges participants in the 2023 study reported encountering. In particular, participants in the earlier study reported concerns about the possibility of a consumers becoming stressed or upset, or felt that some consumers had difficulty understanding RAS-DS. Expert RAS-DS users, while taking a trauma-informed approach, did not shy away from emotional reactions. Instead they were viewed as a valuable opportunity to understand and support consumers to begin to address underlying issues, approaching consumers with openness, sensitivity and a strength-based approach (Tse et al., 2016; Xie, 2013), thereby further facilitating recovery-orientation (Gable & Haidt, 2005). When consumers exhibited difficulties understanding RAS-DS, expert RAS-DS users tailored its administration by providing additional prompts and support as needed. By illustrating how these strategies are implemented in real-world scenarios, this study helps to address the need for more RAS-DS training and support also highlighted in previous research (Honey et al., 2023).

The strategies described in this study are consistent with shared decision-making, recovery-oriented care and trauma-informed practice. Shared decision-making starts with developing a shared understanding of the needs and priorities of the individual (Montori et al. 2023). Findings in this study align with previous research on RAS-DS that found that through conversations about the RAS-DS after completion, people developed a better understanding of their own recovery, and that workers gained new insights into the priorities and experiences of the person they were working with (Hancock et al., 2015, 2020). Workers in this study strategically used diverse prompting questions and communication skills to support consumers in expressing their values, preferences and thoughts (Aoki et al., 2022; Makoul & Clayman, 2006; Stiggelbout et al., 2012). The strategies also align closely with the principles outlined in a recent review of international guidelines for recovery-oriented mental health services, including “cultivating positive hope, establishing partnerships and collaboration, focusing on person-centeredness, and empowerment” (Subandi et al., 2023, p. 4). Further, they are congruent with four out of the six trauma-informed principles as defined by the Substance Abuse and Mental Health Services Administration (2023), namely safety, trustworthiness and transparency, collaboration and mutuality, and empowerment. The collaborative, communicative, and transparent approaches adopted by expert RAS-DS users are all beneficial in helping to build trust, create safety and minimize fear and sense of powerlessness among consumers (Isobel, 2015; Isobel et al., 2021).

The findings have implications beyond the specific use of RAS-DS. The use of outcome measures has at times been criticized as counter to a recovery-orientated approach (Browne, 2006). However, research suggests that if mental health workers fully embrace and implement outcome measures appropriately, for example, by sharing critical information around measurement purpose and value for the individual, and engaging in discussions after completion, more mental health consumers would view completing outcome measures as personally beneficial (Guthrie et al., 2008). Completing outcome measures has been found to offer consumers the opportunity to be heard (Stedman et al., 1997), to improve dialogue with their workers (Graham et al., 2001) and to personally track their progress (Black et al., 2009). By detailing how RAS-DS can be used in a recovery-oriented and trauma-informed way, this study also provides guidance for mental health workers seeking to implement other self-report outcome measures in such a way. This would not only help facilitate implementation, thus bridging the research-to-practice gap (Gotham, 2004, 2006), but also avoid some of the concerns raised in previous literature that outcome measures could be a pothole in consumers’ recovery process if used in formulaic ways, and only to serve organizational requirements (Lakeman, 2004).

Individual mental health workers, however, can often effect limited changes on their own. It is clear that organizational factors are also likely to affect the use of RAS-DS as a collaborative tool. For example, in a previous survey study, mental health workers reported that their use of RAS-DS for collaboration was influenced by such issues as whether it was part of mandatory or standard data collection processes and pathways, the volume of other assessment requirements, provision of training, whether their job requirements allowed enough time to use RAS-DS, and awareness and understanding of RAS-DS as a tool for supporting recovery amongst colleagues (Honey et al., 2023). More than three-quarters of people who mentioned organizational support and policies cited these as barriers rather than facilitators to collaborative RAS-DS use. All participants in the current study worked in organizations that were supportive of RAS-DS usage and relatively recovery-oriented. For instance, they supported but did not make RAS-DS completion mandatory for consumers, and they allowed workers the freedom to use their judgement and devote time to using RAS-DS collaboratively. This, however, is not always the situation, as past research has found that data from outcome measures are often used more for service evaluations, rather than for the best interests for consumers (Johnston & Gowers, 2005; Norman et al., 2014). Hence, it is evident that on top of mental health workers’ own active effort in being recovery-oriented, organizations likewise need to prioritize the fostering of recovery-oriented practice within their systems and structures.

### Limitations and Implications for Future Research

This study focuses solely on mental health workers' perspectives on the approaches they use to facilitate collaboration, without exploring consumers' viewpoints. While consumers have given positive feedback about using RAS-DS with mental health workers (Hancock et al., 2015), future research should investigate consumers' perspectives on which strategies used by their workers are most effective in fostering collaboration. As a small, qualitative study, findings must be interpreted with care and may not be representative of the viewpoints of all mental health workers who use RAS-DS as a tool for collaboration. For example, participants in this study are from only two different countries (Australia and Canada). Thus, how mental health workers in other countries use RAS-DS differently is a topic for future research. Another limitation is that only one participant had used RAS-DS within an inpatient setting. This may be due to the use of RAS-DS in these settings being relatively more unusual. As a result, future research should specifically target practice in this setting to examine whether different strategies are required.

### Conclusions and Implications for Practice

The strategies highlighted in this study demonstrate how mental health workers can use RAS-DS as a tool to facilitate collaboration and person-centred recovery planning. While other tools and methods may serve a similar purpose, the increasingly widespread use of RAS-DS as an outcome measure provides an ideal opportunity to efficiently incorporate this. The expert insights reported here provide guidance for mental health workers regarding the actions and approaches they can employ to implement recovery-focused self-report outcome measures, including RAS-DS, in ways that enhance their own recovery-oriented practice, as well as boosting the engagement and participation of consumers. Further, findings indicate the importance of organizational systems and structures that enable workers to employ these tools flexibly and in ways that best suit the individuals they are working with.
